# Automatic Osteoporosis Screening System Using Radiomics and Deep Learning from Low-Dose Chest CT Images

**DOI:** 10.3390/bioengineering11010050

**Published:** 2024-01-02

**Authors:** Xiaoyu Tong, Shigeng Wang, Jingyi Zhang, Yong Fan, Yijun Liu, Wei Wei

**Affiliations:** Department of Radiology, First Affiliated Hospital of Dalian Medical University, Dalian 116014, Chinawangshigeng9855@163.com (S.W.); fyfan_yong@163.com (Y.F.);

**Keywords:** bone mineral density, osteoporosis, deep learning, tomography, X-ray computed, radiomics

## Abstract

Objective: Develop two fully automatic osteoporosis screening systems using deep learning (DL) and radiomics (Rad) techniques based on low-dose chest CT (LDCT) images and evaluate their diagnostic effectiveness. Methods: In total, 434 patients who underwent LDCT and bone mineral density (BMD) examination were retrospectively enrolled and divided into the development set (*n* = 333) and temporal validation set (*n* = 101). An automatic thoracic vertebra cancellous bone (TVCB) segmentation model was developed. The Dice similarity coefficient (DSC) was used to evaluate the segmentation performance. Furthermore, the three-class Rad and DL models were developed to distinguish osteoporosis, osteopenia, and normal bone mass. The diagnostic performance of these models was evaluated using the receiver operating characteristic (ROC) curve and decision curve analysis (DCA). Results: The automatic segmentation model achieved excellent segmentation performance, with a mean DSC of 0.96 ± 0.02 in the temporal validation set. The Rad model was used to identify osteoporosis, osteopenia, and normal BMD in the temporal validation set, with respective area under the receiver operating characteristic curve (AUC) values of 0.943, 0.801, and 0.932. The DL model achieved higher AUC values of 0.983, 0.906, and 0.969 for the same categories in the same validation set. The Delong test affirmed that both models performed similarly in BMD assessment. However, the accuracy of the DL model is 81.2%, which is better than the 73.3% accuracy of the Rad model in the temporal validation set. Additionally, DCA indicated that the DL model provided a greater net benefit compared to the Rad model across the majority of the reasonable threshold probabilities Conclusions: The automated segmentation framework we developed can accurately segment cancellous bone on low-dose chest CT images. These predictive models, which are based on deep learning and radiomics, provided comparable diagnostic performance in automatic BMD assessment. Nevertheless, it is important to highlight that the DL model demonstrates higher accuracy and precision than the Rad model.

## 1. Introduction

Osteoporosis, a commonly occurring musculoskeletal disease, is characterized by a decrease in bone mineral density (BMD) and damage to the microstructure of bone tissue, leading to heightened bone fragility and an increased risk of fractures [1]. Often termed a “silent disease”, osteoporosis typically exhibits no discernible signs or symptoms until fractures manifest [2]. Notably, osteoporosis-related fractures are the primary cause of morbidity and mortality in the elderly. It is estimated that globally, approximately 9 million new cases of osteoporosis-related fractures occur annually, leading to a substantial burden on public health systems [3,4]. Given these circumstances, it is imperative to prioritize early warning and screening for osteoporosis.

Radiomics (Rad), a quantitative technique that utilizes high-throughput radiomics features, has provided substantial evidence in assessing diseases. Specifically, it has been shown that radiomics can effectively extract BMD information from thoracic vertebrae within chest CT images, enabling the provision of quantitative heterogeneity measures [5]. This approach holds promise for opportunistic and preventive osteoporosis screening, as it eliminates the need for additional costs and radiation exposure. In addition, there is growing concern regarding the radiation risks associated with CT scans, given the increasing utilization of CT imaging and the public’s heightened awareness of radiation protection [6,7]. Low-dose chest CT (LDCT), particularly with a tube voltage of 80 kVp, has been widely applied in clinical practice for lung cancer screening among the high-risk population, as well as routine physical examination [8,9]. However, it is worth noting that modifying the tube voltage setting can potentially impact the stability of the radiomics model [10,11,12]. To the best of our knowledge, the Rad model of BMD assessment based on 80 kVp images has not been well established.

Recently, the field of artificial intelligence has witnessed a surge of interest in deep learning (DL) techniques. DL utilizes deep convolutional neural networks (CNN) to automatically extract high-dimensional features from CT images, enabling end-to-end learning without requiring manual feature extraction [13,14]. DL has exhibited remarkable performance in image analysis and has proven advantages in differentiating between benign and malignant vertebral compression fractures [15]. Although both Rad and DL methods have demonstrated promising diagnostic capabilities in relevant aspects, there exists a dearth of studies comparing their performance in BMD assessment based on chest LDCT images, especially 80 kVp CT images. Can the novel deep learning network surpass traditional radiomics models in accurately diagnosing bone density? 

It is worth noting that both Rad and DL methods require manual delineation of the region of interest, which can be a burdensome workload for radiologists and may introduce observer variability that can impact image analysis. Fortunately, advancements in deep learning architectures have enabled the development of automatic segmentation models that can mitigate these challenges and provide satisfactory segmentation results [16,17]. Therefore, this study had dual objectives. Firstly, we endeavored to train an automatic segmentation model using VB-Net architecture specifically for thoracic vertebra cancellous bone (TVCB). Secondly, we aimed to develop and compare the diagnostic performance of two predictive models—a deep learning-based model (DL model) and a radiomics-based model (Rad model)—for BMD assessment based on low-dose chest CT images acquired at 80 kVp. We hypothesize that the novel DL model may outperform traditional Rad models in accurately assessing bone mineral density.

## 2. Materials and Methods

This retrospective study received approval from the Ethics Committee, which also waived the requirement for informed patient consent (IRB No. PJ-KS-KY-2023-276).

### 2.1. Study Population

**Figure 1:**
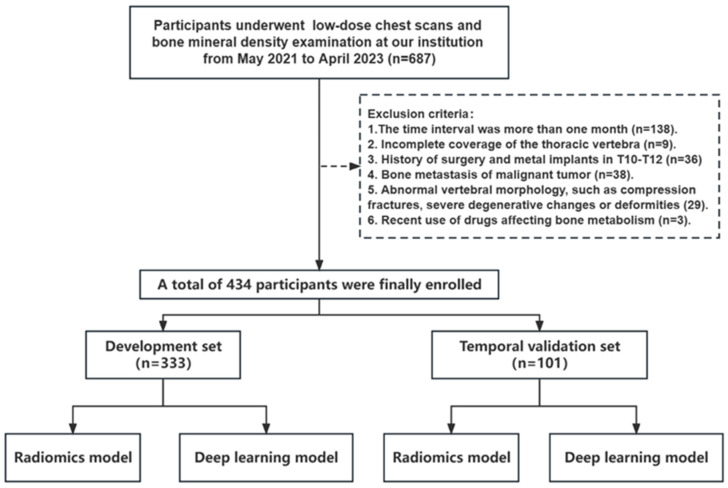
A detailed flowchart of patient enrollment.

**Figure 2:**
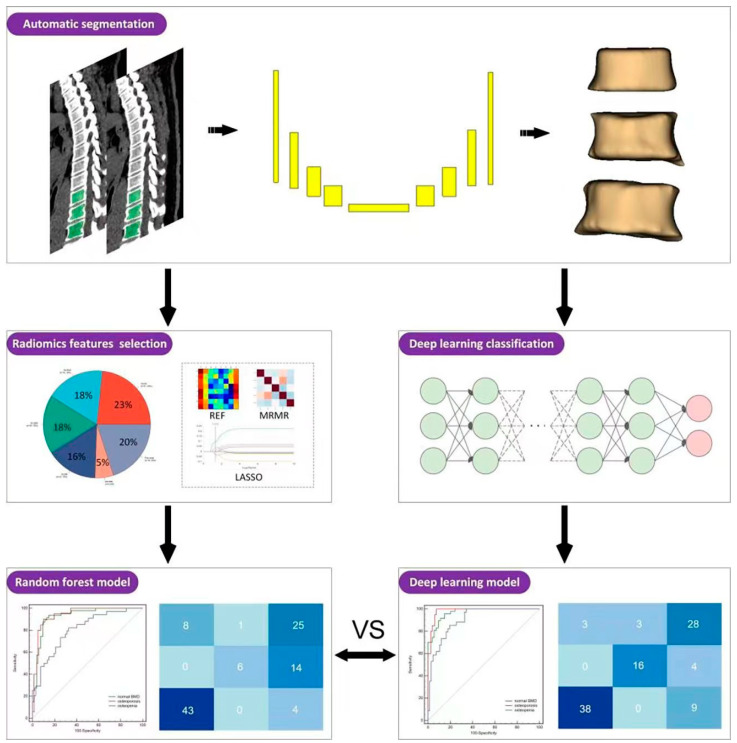
The overall flowchart of this study. The construction of the radiomics model utilizes a random forest classifier, while the deep learning model adopts the Res-Net architecture. REF, Recursive Feature Elimination; MRMR, Minimum Redundancy Maximum Relevance; LASSO, the Least Absolute Shrinkage and Selection Operator.

A total of 687 patients who underwent chest LDCT scans and BMD examination were retrospectively retrieved from the picture archiving and communication system from May 2021 to April 2023. Patients with the following conditions were excluded: (1) the time interval between LDCT and BMD was more than one month (*n* = 138); (2) the scanning range failed to cover the required thoracic vertebra (*n* = 9); (3) a history of surgery and metal implants in the lower thoracic vertebrae (*n* = 36); (4) bone metastasis of malignant tumors (*n* = 38); (5) abnormal vertebral morphology in the lower thoracic vertebrae, such as compression fracture, severe degenerative changes or deformities (*n* = 29); and (6) recent use of drugs affecting bone metabolism (*n* = 3). Eventually, 434 patients were enrolled and divided into a development set (*n* = 333, examined between May 2021 and June 2022) and a temporal validation set (*n* = 101, examined between July 2022 and April 2023) according to the examination time. This development set was utilized for training automatic segmentation models as well as BMD assessment models. During the training of the BMD assessment models (Rad and DL model), the development set was randomly partitioned into two subsets for BMD assessment models training and internal evaluation, with 80% allocated for the internal training set and the remaining 20% for the testing set. The temporal validation set was used to evaluate the performance of all models. A detailed enrollment flowchart is shown in Figure 1, and the overall workflow of this study is illustrated in Figure 2. The automatic segmentation framework construction, Rad model, and DL model were developed and validated on the uAI Research Portal V1.1 (United Imaging Intelligence, Co., Ltd. (Shanghai, China)). The design of uAI Research Portal architecture takes a modular and layered approach [18]: (1) The lower level is composed of hardware drivers, such as graphics processing unit (GPU) accelerated using NVIDIA CUDA, and cloud servers, such as Amazon web services (AWS); (2) At the middle level, there is an application programming interface (API), primarily Python and C++, contributing a range of algorithms (e.g., segmentation, classification); (3) the higher level presents build blocks to the end users for domain-specific analysis.

### 2.2. Image Acquisition and BMD Assessment

All CT images were acquired on a 256-row CT scanner (Revolution CT, GE HealthCare, Milwaukee, WI, USA). The chest LDCT scans were acquired using a low tube voltage of 80 kV, smart mA (noise index: 10, 50–400 mA), rotation speed of 0.5 s/rot, detector width of 80 mm, pitch of 0.992, and scanning slice thickness and slice interval of 5 mm. The scan coverage started from the lung apexes to 2 cm below the diaphragm. All images were reconstructed using the standard kernel, adaptive statistical iterative reconstruction-Veo (ASIR-V) at 40% strength, and reconstruction thickness and interval of 1.25 mm. 

BMD examinations were performed using a standardized protocol following the manufacturer’s guidelines for the quantitative computed tomography (QCT) workstation. The details of the QCT scanning protocol can be found in Supplementary S1. Patient abdominal data were transferred to a QCT Pro workstation (version 6.1, Mindways Software, Inc. (Austin, TX, USA)), and BMD measurements were taken at two consecutive vertebral bodies (L1 and L2). Compared to conventional methods, QCT measures volumetric BMD, reflecting BMD in different regions (trabecular and cortical) of the skeleton. This gives QCT an advantage in assessing osteoporosis severity, guiding treatment strategies, and monitoring treatment efficacy. According to clinical guidelines for BMD assessment [19], osteoporosis was defined as a BMD below 80 mg/cm^3^, osteopenia as a BMD between 80 and 120 mg/cm^3^, and normal status as a BMD above 120 mg/cm^3^. For this study, the diagnostic performance was analyzed through the construction of receiver operating characteristic (ROC) curves, employing QCT data as the diagnostic standard. The ROC curve assesses a classification or diagnostic model’s performance by plotting the true positive (sensitivity) and false positive rate (1-specificity) against various thresholds [20]. This provides an overview of the model’s performance in predicting bone status.

### 2.3. TVCB Auto-Segmentation Framework and VOI Delineation

Budoff et al. suggested that the cancellous bone of the lower thoracic vertebrae (TVCB), specifically T10–12, closely correlates with lumbar vertebrae in providing information about bone mineral density (BMD), making it a viable target for BMD assessment [21]. Therefore, the volume of interest (VOI) of TVCB was manually delineated on the axial images and carefully avoided vertebral venous plexuses and cortical bone. The boundary was placed along the inner edge of the vertebral cortex. In addition, 100 patients were randomly selected to assess the interobserver repeatability in the manual segmentation, and VOI was independently delineated on CT images by two readers (W. Wei and Y. Liu, with 5 and 12 years of experience in musculoskeletal radiology, respectively). The Dice similarity coefficient (DSC) was employed to assess the consistency of inter-observer segmentation. If a satisfactory agreement was achieved, the junior radiologist would complete the remaining cases under the supervision of the senior radiologist.

For the auto-segmentation framework, we trained a cascade model with two VB-Nets based on the coarse-to-fine principle, including a coarse-scale segmentation network for rapidly locating the target area and a fine-scale segmentation network for precisely delineating target and optimization. The detailed architecture of the VB-Net is shown in Supplementary Figure S1. In pre-processing, it was normalized by subtracting the window level (WL: 100) and dividing by the window width (WW: 300). For training the coarse-scale segmentation network, global sampling was used. The images were resampled to 3 × 3 × 3 mm using B-Spline interpolation. In the fine-scale segmentation network, images were resampled to crop high-resolution local images with a resampling voxel size of 1 × 1 × 1 mm, and mask sampling was used. The learning rate was 1 × 10^−4^, the batch size was 8, the number of epochs was set to 1001, and the optimizer was Adam. We used the focal loss function to monitor the convergence of the training model and optimize the network. The detailed settings of the coarse-scale segmentation network are given in Supplementary S2. DSC and volume difference (VD) were used to evaluate the segmentation performance of the model. The DSC coefficient is a measure of similarity between the segmentation results and the reference criteria. Its calculation method is based on the overlapping area between the segmentation result and the reference standard. The VD was defined as the absolute value of the manually segmented volume minus the automatically segmented volume.

### 2.4. Radiomics Model Construction

After establishing the auto-segmentation model, the model was used for automatic cancellous bone segmentation in the development and temporal validation sets.

#### 2.4.1. Radiomics Features Extraction

All images were normalized using Z-score and resampled, the voxel spacing to 1 × 1 × 1 mm using B-Spline interpolation, and the image gray level was discretized with 25 binwidth. Z-score normalization is a widely utilized technique for standardizing data to make it comparable across different features. This is accomplished by subtracting the mean from each data point and dividing it by the standard deviation of the feature’s data within the given sample. A total of 90 features in six categories were extracted from the original images, including first-order features and texture features. Details of the extracted radiomics features are provided in Supplementary Table S1. 

#### 2.4.2. Features Selection and Model Construction

The development set was randomly divided into the internal training and testing sets at a ratio of 8:2. The Z-score normalization was conducted to pre-process the features and ensure the comparability between the data before the feature selection and Rad model construction. A step-wise feature selection strategy was used to determine the optimal features (Supplementary S3). 

Finally, random forest was performed to establish a three-classification model to distinguish osteoporosis, osteopenia, and normal BMD. Random forest is a widely used ensemble technique in radiomics classification tasks. It is based on a collection of decision trees, forming a “forest”, and incorporates random feature selection and bootstrap sampling during training and prediction. The ROC curve was conducted to evaluate the efficacy of the Rad model in diagnosing osteoporosis, osteopenia, and normal BMD. The area under the receiver operating characteristic curve (AUC), sensitivity, specificity, precision, and accuracy were calculated to evaluate the performance of training, internal test, and temporal validation set.

### 2.5. Deep Learning Network Construction

**Figure 3:**
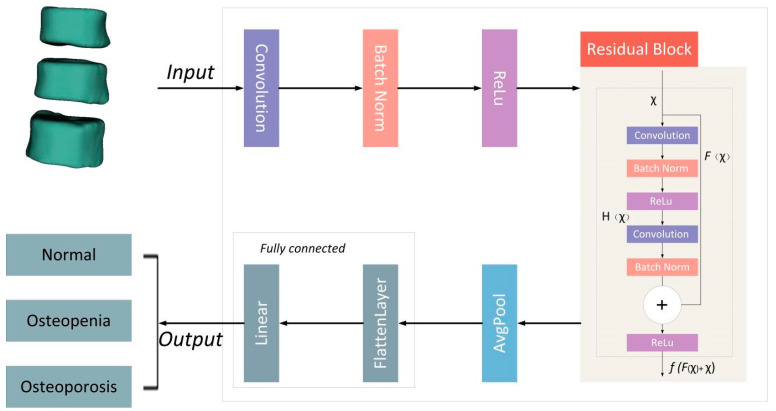
The Residual Network Structure.

The DL model was trained using the residual network (Res-Net), which integrates residual learning to prevent gradient dispersion and precision loss in deep networks, achieving enhanced accuracy as the network depth increases [22]. The Res-Net is composed of four simple residual blocks, which enable the network to learn more efficiently and effectively; each residual block consists of two convolutional layers followed by a skip connection, which can effectively learn both low-level and high-level features simultaneously. During the training process, all images were resampled with voxel spacing of 1 × 1 × 1 mm and normalized by min-max normalization. Figure 3 shows the detailed architecture of the Res-Net. The batch size was set to 8, and the IO threads were set to 4. The focal loss function and Adam optimizer were used to monitor the convergence of the model with an initial learning rate of 1 × 10^−4^, and the “step” learning rate strategy was applied to accelerate convergence. The diagnostic performance of the deep learning classification model was evaluated on the internal test and temporal validation sets using ROC analysis. 

### 2.6. Statistical Analysis

SPSS version 24.0 (IBM Corp., Armonk, NY, USA) and MedCalc version 20.022 (MedCalc Ltd., Ostend, Belgium) were used for statistical analysis. The data were tested for normality using the Kolmogorov–Smirnov test, and continuous variables were expressed as mean ± standard deviation or medians (25–75th percentile). The chi-square test was used for gender and bone status distribution in development and temporal validation sets. An independent sample *t*-test was used to test the age difference between the development and the temporal validation sets. The DeLong test was used to assess the difference in diagnostic performance between the Rad model and the DL model. The clinical application value of the Rad model and the DL model was evaluated in the temporal validation set by constructing decision curve analysis (DCA).

## 3. Results

### 3.1. Participant Demographics

A total of 434 patients were enrolled in the study, including 333 patients in the development set (mean age: 62.89 ± 11.55 years) and 101 patients in the temporal validation set (mean age: 60.76 ± 10.41 years). In both sets, there were no significant differences in the distributions of age, gender, and BMD distribution. The detailed demographic characteristics are shown in Table 1.

### 3.2. Automatic Segmentation Model

**Figure 4:**
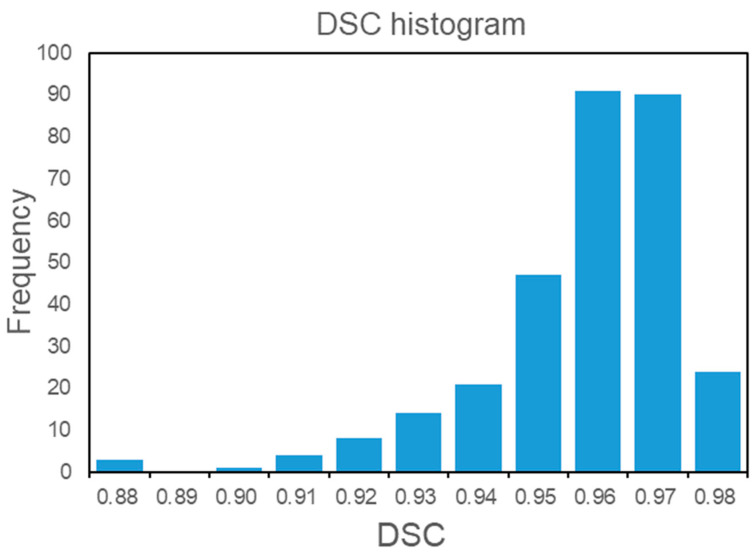
Histogram of the DSC. DSC, Dice similarity coefficient.

The cancellous bone segmentation was in good agreement between the two observers, with a mean DSC of 0.96 ± 0.02. The automatic segmentation model demonstrated excellent performance with a mean DSC of 0.96 ± 0.02 in the temporal validation set. The detailed distribution of DSC is shown in Figure 4. The VD did not exceed 1 cm^3^ with a mean of 0.50 (0.17, 0.69). The segmentation performance of TVCB for different BMD populations is illustrated in Table 2.

### 3.3. The Comparison of the Rad Model and DL Model

In the Rad model, 6 radiomics features were selected, including 1 first-order feature and 5 texture features (Supplementary Table S2). The AUCs in predicting osteoporosis, osteopenia, and normal BMD were 0.919, 0.873, and 0.976, respectively, in the internal test set. In the temporal validation set, the AUCs were 0.943, 0.801, and 0.932, respectively.

As for the DL model, the AUCs in predicting osteoporosis, osteopenia, and normal BMD were 0.942, 0.866, and 0.972, respectively, in the internal test set. In the temporal validation set, the AUCs were 0.983, 0.906, and 0.969, respectively.

**Figure 5:**
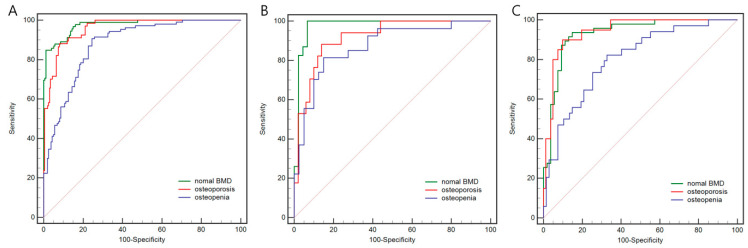
The receiver operating characteristic curves of the radiomics model on the internal training (**A**), the internal testing set (**B**), and the temporal validation set (**C**). The AUC values of the radiomics model on the internal training set for osteoporosis, osteopenia, and normal BMD were 0.959, 0.881, and 0.977, respectively. As for the internal testing set, these values were 0.919, 0.873, and 0.976, respectively. As for the temporal validation set, these values were 0.943, 0.801, and 0.932, respectively. The red, blue, and green lines represent predicted osteoporosis, osteopenia, and normal BMD, respectively. AUC, area under the receiver operating characteristic curve; BMD, bone mineral density.

**Figure 6:**
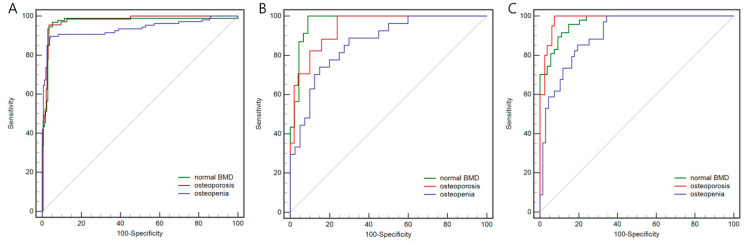
The receiver operating characteristic curves of the deep learning model on the internal training (**A**), the internal testing set (**B**), and the temporal validation set (**C**). The AUC values of the radiomics model on the internal training set for osteoporosis, osteopenia, and normal BMD were 0.975, 0.936, and 0.972, respectively. As for the internal testing set, these values were 0.942, 0.866, and 0.972, respectively. As for the temporal validation set, these values were 0.983, 0.906, and 0.969, respectively. The red, blue, and green lines represent predicted osteoporosis, osteopenia, and normal BMD, respectively. AUC, area under the receiver operating characteristic curve; BMD, bone mineral density.

**Figure 7:**
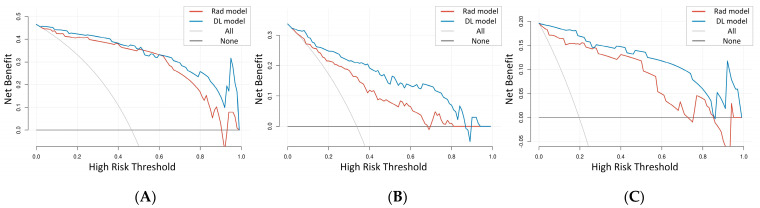
DCAs of the Rad model and DL model in the temporal validation set. (**A**) DCA of the Rad model and DL model in predicting abnormal BMD. (**B**) DCA of the Rad model and DL model in predicting osteopenia. (**C**) DCA of the Rad model and DL model in predicting osteoporosis. Rad model, radiomics model; DL model, deep learning model; DCA, decision curve analysis; BMD, bone mineral density.

The two models achieved similar performance in distinguishing osteoporosis, osteopenia, and normal BMD for the temporal validation set, with no significant difference demonstrated by the DeLong test. The results of more detailed metrics are summarized in Table 3, and the ROC curves are shown in Figure 5 and Figure 6. DCA showed that the DL model had a higher net benefit than the Rad model across the majority of the range of reasonable threshold probabilities in the temporal validation set, indicating that the DL model has good clinical utility (Figure 7). 

Furthermore, we compared the performance of the proposed method with several benchmark methods. The comparison reveals that our DL model demonstrates superior performance in detecting osteoporosis, osteopenia, and normal BMD (Table 4).

## 4. Discussion

In this study, we developed an automatic TVCB segmentation model using the VB-Net network architecture and a coarse-to-fine cascade training strategy based on 80 kVp chest LDCT images. The model achieved segmentation accuracy comparable to that of manual depiction, with mean DSC surpassing 0.90. In addition, we compared the classification performance between the Rad and DL models for BMD assessment. The AUCs of the Rad and DL models were 0.943 and 0.983 for predicting osteoporosis, 0.801 and 0.906 for predicting osteopenia, and 0.932 and 0.969 for predicting normal BMD in the validation set, respectively. The Delong test showed that for diagnostic performance, there was no statistically significant difference between the Rad and DL models. However, the DL model demonstrated superior sensitivity, specificity, precision, and overall accuracy in evaluating various performance metrics compared to the traditional Rad model. In addition, the end-to-end learning strategy employed in the DL model eliminated intermediate steps such as Rad model data pre-processing, feature extraction, and classifier selection, which reduces human intervention and improves the efficiency of model construction and the objectivity of the results.

LDCT is primarily accomplished by reducing the tube current or tube voltage. As the radiation dose is directly proportional to the square of the tube voltage, reducing the tube voltage can effectively decrease the radiation dose [26]. This is particularly advantageous for the Asian population, which typically has smaller body sizes. Lower tube voltage scans do not significantly compromise diagnostic confidence but provide more cost-effective and radiation-dose-efficient imaging for patients [27].

To the best of our knowledge, no attempt has been made to establish automatic segmentation of TVCB on LDCT using 80 kVp. Previous segmentation models have been constructed using 120 kVp images in a complex or error-prone manner. Chen et al. initially used CNN networks to identify the entire thoracic vertebrae and subsequently applied an erosion algorithm to remove the bone cortex [24]. However, the working process of this method to obtain TVCB was complex. Wang et al. used a fixed-size cylindrical shape to identify TVCB, leading to incomplete segmentation in cancellous bone and introducing bias in the BMD assessment [28]. In our study, we employed the VB-Net to construct an automatic segmentation model for TVCB. The VB-Net is a modified version of CNN that incorporates a bottleneck structure in place of convolutional, normalization, and activation layers. This modification not only reduces the number of model parameters but also improves inference efficiency and robustness [28]. The VB-Net has been demonstrated to produce satisfactory segmentation results, with established applications in cervical and lung cancer segmentation [29,30]. Additionally, the uneven distribution of BMD in the thoracic vertebrae can reduce the sensitivity of osteoporosis assessment if the entire vertebrae are segmented. However, some researchers found that utilizing the lower thoracic vertebrae (T10–12) for BMD assessment yields high levels of accuracy and repeatability [21]. Therefore, we used a cascade approach utilizing VB-Net in this work. The 10-12th vertebrae were initially identified at a “coarse” resolution to achieve accurate spatial localization, followed by the detailed delineation of bony cortex and cancellous bone at a “fine” resolution. It is worthwhile to emphasize the advantages of our method, as it efficiently and accurately identifies the TVCB in chest LDCT images. Our method achieved DSC results exceeding 0.90 in the temporal validation set. 

Radiomics encompasses the extraction of high-dimensional tissue data from medical images, which can be further integrated with machine learning techniques to establish radiomics signatures. The radiomics features extracted from TVCB can reflect the transformation of bone microstructure and accurately assess BMD [31]. Notably, Chen et al. pointed out that the performance of radiomics in assessing BMD significantly decreased in the external validation set, with a 20% lower accuracy compared to the internal validation set [24]. This phenomenon occurs because the stability of radiomics features depends on image acquisition parameters. Altering factors such as tube voltage or slice thickness can indeed impact the effectiveness of existing radiomics models [32]. Therefore, we deem it imperative to establish a novel Rad model in 80 kVp chest CT images for BMD assessment. To ensure the stability of the feature selection process and the generalization ability of the Rad model, we employed a step-wise feature selection strategy to select 6 highly effective features. Encouragingly, these 6 features have been closely related to bone quantity, microstructure, and loss in relevant studies [23,25]. Our Rad model could provide valuable information in BMD assessment and demonstrate comparable or superior performance compared to recent research results.

Deep learning has emerged as a highly promising approach for achieving accurate diagnostic outcomes in medical imaging. Recent advancements in artificial intelligence have been crucial in driving this progression. Mehdi et al. developed a DL model that was capable of distinguishing tumor invasiveness, achieving accuracy comparable to pathology results [33]. Li et al. developed a DL model based on CT images using Res-Net, achieving faster convergence and high accuracy in diagnosing vertebral fractures [34]. Kitamura et al. [35] discovered that Res-Net convolutional neural networks demonstrated strong performance in ankle fracture detection with small sample sizes. Therefore, we selected Res-Net to build a three-classification BMD assessment model. The core concept of Res-Net is to learn residuals, which involves the network learning the difference between inputs and outputs. To tackle the challenges of vanishing and exploding gradients in training deep neural networks, Res-Net introduces “residual blocks” [34]. These blocks enable the network to efficiently capture the disparity between input and output through shortcut connections, resulting in faster convergence and improved accuracy [36]. With this innovative network architecture, Res-Net empowers models to train deeper neural networks, effectively addressing complex visual tasks like image classification, object detection, and semantic segmentation.

In our study, the DL model utilized automatic segmentation of TVCB as an input, eliminating the need for time-consuming, manually segmented regions of interest. Furthermore, the DL model enabled the extraction and analysis of high-level semantic features in an end-to-end manner, facilitating the automatic learning of pertinent and robust features without human intervention. Consequently, the overall approach mitigated human bias arising from artificial features. Our DL model yielded satisfactory outcomes in BMD assessment.

Previous studies have compared the performance of Rad and DL models across various tasks. Mehdi et al. [33] discovered that the Rad model outperformed the DL model in predicting malignancy of pulmonary nodules from chest LDCT images. Li et al. [37] observed that their DL model outperformed the Rad model in classifying molecular subtypes of diffuse gliomas. In our study, we developed Rad and DL models based on relatively large samples. To the best of our knowledge, this is the first study to investigate and compare the performance of DL networks against traditional Rad models in assessing BMD. The main findings of our study demonstrate that the novel DL model outperformed the traditional Rad model in the precise assessment of BMD. The DL model exhibited enhanced sensitivity, specificity, precision, and overall accuracy across various performance metrics relative to the traditional Rad model. Zhou et al. [37] obtained similar results when distinguishing between benign and malignant breast lesions using Rad and DL models. In the Rad model, it is necessary to determine the most suitable features for the BMD assessment task in advance. In contrast, the DL model does not require predefined features and can automatically determine the nuanced features of the target task with almost no human intervention, ensuring objectivity and efficient classification performance. Consequently, our results indicate that deep learning has the potential to serve as a diagnostic tool for BMD assessment in clinical practice. The improved performance of the DL model can provide enhanced diagnostic accuracy, thus leading to better clinical decision-making and improving patient outcomes. By accurately predicting BMD status, clinicians can identify individuals at high risk of fractures and tailor intervention strategies accordingly. This approach can lead to early interventions that prevent or mitigate the progression of bone diseases, ultimately improving patient outcomes and reducing healthcare costs. Furthermore, the successful application of DL networks in assessing BMD highlights the potential for similar approaches in other medical domains. 

This study has some limitations. Firstly, our proposed Rad and DL models were developed using chest LDCT images acquired from a single center, which may restrict their applicability of the models to the LDCT in other institutions. Additionally, while residual networks have shown significant success in enhancing performance, the architecture can also suffer from black-box effects stemming from the complexity caused by convolutional layers and non-linear activation functions. Finally, the selection of the random forest classifier, although informed by a comprehensive literature review, was not accompanied by comparative analysis against other potential classifiers in our study, which leaves room for exploration regarding optimal classification strategies.

## 5. Conclusions

In conclusion, we developed and evaluated a model for automatic TVCB segmentation using 80 kVp chest LDCT images, which laid the foundation for future fully automated BMD assessment programs. In addition, we developed deep learning-based and radiomics-based predictive models, which provided similar excellent diagnostic performance in BMD assessment. Nevertheless, it is important to highlight that the deep learning model demonstrates higher accuracy and precision compared to the radiomics model. Future research should investigate whether variations in CT scan parameters would affect the performance of DL models in assessing bone mineral density.

## Figures and Tables

**Table 1:** Participant demographics.

Characteristic	Development Set	Temporal Validation Set	*p*-Value
All (*n*)	333	101	
Male (*n*)	170	57	
Female (*n*)	163	44	0.404
All (years)	62.89 ± 11.55	60.76 ± 10.41	0.098
Male (years)	65.37 ± 10.37	62.60 ± 9.14	0.073
Female (years)	60.30 ± 12.17	58.39 ± 11.54	0.350
Osteoporosis (*n*)	84	20	
Osteopenia (*n*)	134	34	
Normal BMD (*n*)	115	47	0.094

Data are presented as the number of patients except for mean ± standard deviation for age.

**Table 2:** The Dice similarity coefficient and volume difference of manual and automatic segmentation.

Category	DSC	VD (cm^3^)
All	0.96 ± 0.02	0.50 (0.17, 0.69)
Osteoporosis	0.97 ± 0.01	0.44 (0.09, 0.68)
Osteopenia	0.96 ± 0.02	0.53 (0.19, 0.63)
Normal BMD	0.96 ± 0.02	0.50 (0.18, 0.80)

DSC, Dice similarity coefficient; VD, volume difference; BMD, bone mineral density.

**Table 3:** Overall performance of BMD assessment for the Rad model and DL model.

Model	Set	Category	AUC	95%CI	Sensitivity (%)	Specificity (%)	Precision (%)	Accuracy (%)
Rad Model	Internal training set	Osteoporosis	0.959	0.927–0.979	88.1	92.0	88.9	
		Osteopenia	0.881	0.835–0.917	90.7	75.5	67.3	
		Normal BMD	0.977	0.95–0.991	84.8	98.9	96.3	
		Overall						79.0
	Internal test set	Osteoporosis	0.919	0.826–0.971	88.2	86.0	71.4	
		Osteopenia	0.873	0.769–0.942	81.5	85.0	60.0	
		Normal BMD	0.976	0.906–0.998	100.0	93.2	90.0	
		Overall						70.2
	Temporal validation set	Osteoporosis	0.943	0.878–0.979	90.0	90.1	85.7	
		Osteopenia	0.801	0.709–0.874	82.4	67.2	58.1	
		Normal BMD	0.932	0.864–0.972	93.6	85.2	84.3	
		Overall						73.3
DL Model	Internal training set	Osteoporosis	0.975	0.948–0.990	95.5	96.5	87.7	
		Osteopenia	0.936	0.900–0.962	89.7	95.6	93.2	
		Normal BMD	0.972	0.944–0.988	96.7	94.8	95.6	
		Overall						92.5
	Internal test set	Osteoporosis	0.942	0.857–0.985	100.0	76.0	75.0	
		Osteopenia	0.866	0.760–0.937	74.1	85.0	71.4	
		Normal BMD	0.972	0.900–0.997	100.0	90.9	87.0	
		Overall						77.6
	Temporal validation set	Osteoporosis	0.983	0.935–0.998	100.0	92.6	84.2	
		Osteopenia	0.906	0.831–0.955	85.3	80.6	68.3	
		Normal BMD	0.969	0.914–0.993	95.7	85.2	92.7	
		Overall						81.2

DL, deep learning; Rad, radiomics; AUC, area under the receiver operating characteristic curve; CI, confidence interval; BMD, bone mineral density.

**Table 4:** The performance of our proposed method is compared with several benchmark methods.

Authors(methods)	Key Findings	AUC	Sensitivity (%)	Specificity (%)	Accuracy (%)
Xue et al. (Radiomics) [23]	Detecting abnormal BMD	0.944	95.8	-	-
	Detecting osteoporosis	0.866	83.3	-	-
Chen et al. (Radiomics) [24]	Detecting abnormal BMD	0.960	93.0	89.0	91.0
	Detecting osteoporosis	0.980	95.0	93.0	94.0
Wang et al. (Radiomics) [25]	Detecting osteoporosis	0.914	90.7	75.0	89.8
Ours (Radiomics)	Detecting abnormal BMD	0.932	93.6	85.2	73.3
	Detecting osteopenia	0.801	82.4	67.5	
	Detecting osteoporosis	0.943	90.0	90.1	
Yang et al. (Deep learning) [5]	Detecting osteopenia	0.831	73.6	80.5	-
	Detecting osteoporosis	0.972	95.6	88.0	
Ours (Deep learning)	Detecting abnormal BMD	0.969	95.7	85.2	81.2
	Detecting Osteopenia	0.906	85.3	80.6	
	Detecting osteoporosis	0.983	100	92.6	

BMD, bone mineral density.

## Data Availability

The raw data supporting the conclusions of this article will be made available by the authors without undue reservation.

## Supplementary Materials

The following supporting information can be downloaded at: https://www.mdpi.com/article/10.3390/bioengineering11010050/s1, Supplementary S1: The BMD scanning protocol and measurement; Supplementary S2: The detailed settings of the coarse-scale segmentation network; Supplementary S3: Radiomic features extraction methods; Figure S1: VB-Net architecture. VB-Net is a variant segmentation network structure of V-Net that utilizes a bottleneck structure (B stands for bottleneck) instead of the convolution, normalization, and activation layers within the Down Block and Up block. A bottleneck structure in a neural network has fewer neurons than its adjacent layers, which helps compress feature representations to fit in the available vector space. The bottleneck structure consists of three convolutional layers. The first and third convolutional layers use the unit convolution kernel and match the dimensions of the preceding and succeeding layers, respectively. The second convolution layer performs spatial convolution on the feature image that has been reduced in dimension by the first convolution layer. This reduction in dimensionality helps reduce the number of model parameters, leading to increased efficiency; Table S1: Radiomics features extracted from original images; Table S2: Selected features for constructing radiomics model.
