# Supplementary material for: Automatic Osteoporosis Screening System Using Radiomics and Deep Learning from Low-Dose Chest CT Images
Source: Bioengineering (Basel). 2024 Jan 2;11(1):50. doi: 10.3390/bioengineering11010050 (PMC10813496; doi:10.3390/bioengineering11010050)
Supplement: Supplementary file 1 [file bioengineering-11-00050-s001.zip › bioengineering-2769600-supplementary.pdf]

SUPPLEMENTARY

Supplementary S1**.** The BMD scanning protocol and measurement

All BMD examinations were performed with a standard protocol according to the manufacturer's instructions. The main scan parameters were 120 kV, smart mA (noise index: 10, 50-400mA), scanning field of view of 50 cm, and reconstruction thickness and interval of 1.25 mm. All images were transferred to a dedicated QCT post-processing workstation (Model 4 QCT pro v6.1, Mindways Software, Inc., Austin, USA) for BMD assessment. In addition, a QCT calibration phantom (Mindways Software Inc., Austin, TX, USA) was scanned once a week for accurate asynchronous BMD analysis.

For BMD assessment, the BMDs of L1-2 vertebrae were measured, and the average values were calculated. An oval volume of interest (VOI) was manually placed with a depth of 9 mm, which avoids cortical bone, vertebral venous plexus, and bone islands, covering approximately 2/3 of cancellous bone. According to the clinical diagnostic criteria of BMD assessment, osteoporosis was defined as an average BMD of L1-L2 <80 mg/cm^3, osteopenia as a range of 80-120 mg/cm^3, and normal BMD as >120 mg/cm^3.

Supplementary S2**.** The detailed settings of the coarse-scale segmentation network

The learning rate was 1e-4, batch size was 8, the number of epochs was set to 1001, and the optimizer was Adam. We used the Focal loss function to monitor the convergence of the training model and optimize the network, a variant of the cross-entropy loss function that alleviates the impact of class imbalance on model training by adjusting sample weights.

Supplementary S3**.** Radiomic features extraction methods

A step-wise feature selection strategy was used to determine the optimal features. which sequentially apply three feature selection methods: Recursive Feature Elimination (RFE), Minimum Redundancy Maximum Relevance (mRMR), and the Least Absolute Shrinkage and Selection Operator (LASSO).

RFE iteratively eliminates the least relevant features based on their importance rankings. The mRMR reduces the mutual redundancy between features and retains the largest relevant features. LASSO is a regularization method that minimizes the loss function with a constraint on the L1 norm of the model parameters. By shrinking some feature weights to zero, LASSO achieves feature selection.

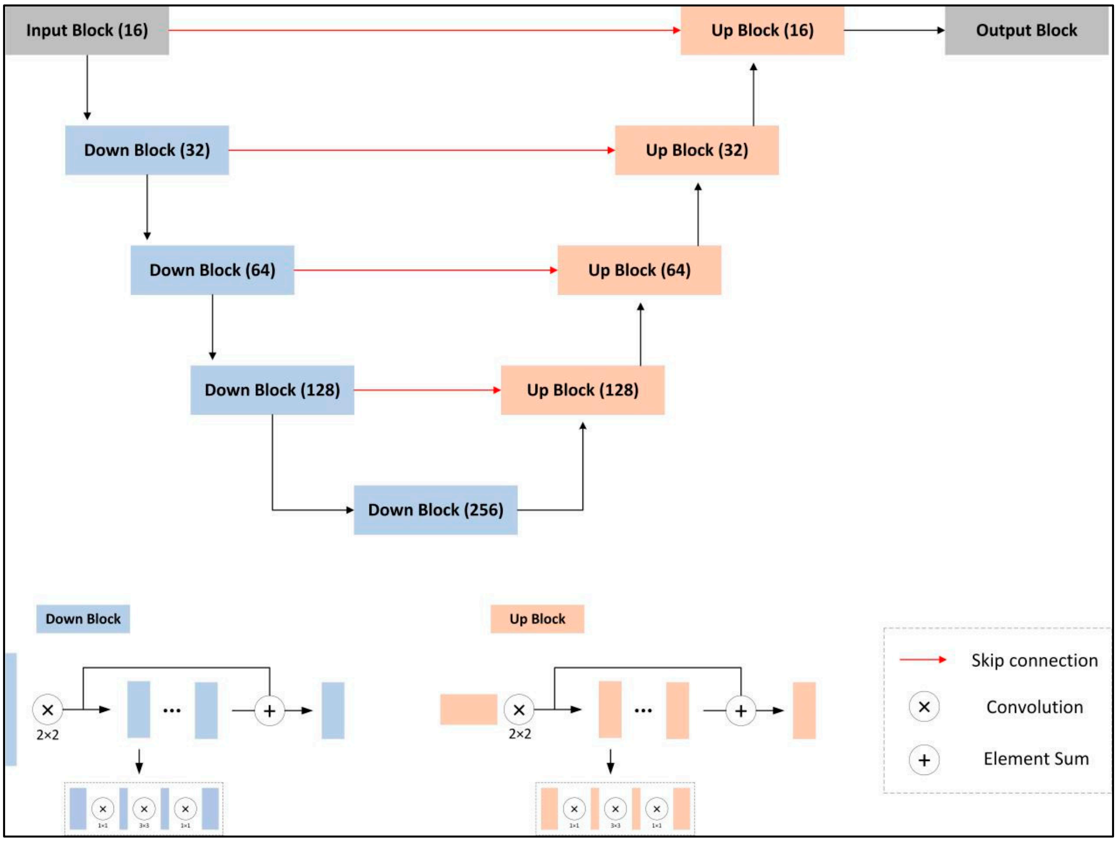

Supplementary Figure S1. VB-Net architecture. VB-Net is a variant segmentation network structure of V-Net that utilizes a bottle-neck structure (B stands for bottle-neck) instead of the convolution, normalization, and activation layers within the Down Block and Up block. A bottleneck structure in a neural network has fewer neurons than its adjacent layers, which helps compress feature representations to fit in the available vector space. The bottleneck structure consists of three convolutional layers. The first and third convolutional layers use the unit convolution kernel and match the dimensions of the preceding and succeeding layers, respectively. The second convolution layer performs spatial convolution on the feature image that has been reduced in dimension by the first convolution layer. This reduction in dimensionality helps reduce the number of model parameters, leading to increased efficiency.

Supplementary Table S1. Radiomics features extracted from original images

Category	Features	Category	Features
	10 Percentile		Gray Level NonUniformity Normalized
	90 Percentile		Gray Level Variance
	Energy		High Gray Level Run Emphasis
	Entropy		Long Run Emphasis
	Interquartile Range		Long Run High Gray Level Emphasis
	Kurtosis		Long Run Low Gray Level Emphasis
	Maximum		Low Gray Level Run Emphasis
	Mean Absolute Deviation	GLRLM	Run Entropy
	Mean		Run Length NonUniformity
First order	Median		Run Length NonUniformity Normalized
	Minimum		Run Percentage
	Range		Run Variance
	Robust Mean Absolute		
	Deviation		Short Run Emphasis
	Root Mean Squared		Short Run High Gray Level Emphasis
	Skewness		Short Run Low Gray Level Emphasis
	Total Energy		Gray Level NonUniformity
	Uniformity		Gray Level NonUniformity Normalized
	Variance		Gray Level Variance
	Autocorrelation		High Gray Level Zone Emphasis
	Joint Average		Large Area Emphasis
	Cluster Prominence	GLSZM	Large Area High Gray Level Emphasis
GLCM	Cluster Shade		Large Area Low Gray Level Emphasis
	Cluster Tendency		Low Gray Level Zone Emphasis
	Contrast		Size Zone NonUniformity
	Correlation		Size Zone NonUniformity Normalized
	Difference Average		Small Area Emphasis

	Difference Entropy		Small Area High Gray Level Emphasis
	Difference Variance		Small Area Low Gray Level Emphasis
	Joint Energy		Zone Entropy
	Joint Entropy		Zone Percentage
	Inverse Variance		Zone Variance
	Maximum Probability		Dependence Entropy
	Sum Entropy		Dependence NonUniformity
	Idn		Dependence NonUniformity Normalized
	Id		Dependence Variance
	Idmn		Gray Level NonUniformity
	Idm		Gray Level Variance
	Imc2	GLDM	High Gray Level Emphasis
	Imc1		Large Dependence Emphasis
	Busyness		Large Dependence High Gray Level Emphasis
	Coarseness		Large Dependence Low Gray Level Emphasis
NGTDM	Complexity		Low Gray Level Emphasis
	Contrast		Small Dependence Emphasis
	Strength		Small Dependence High Gray Level Emphasis
GLRLM	Gray Level NonUniformity		Small Dependence Low Gray Level Emphasis

GLCM=Gray-level Co-occurrence Matrix; NGTDM=Neighborhood Gray Tone Difference Matrix; GLRLM=Gray Level Run Length Matrix; GLSZM=Gray Level Size Zone Matrix; GLDM=Gray Level Dependence Matrix.

Supplementary material Table S2. Selected features for constructing radiomics model

Category	Features
First order	10 Percentile
NGTDM	Contrast
GLDM	Dependence Variance
GLDM	Dependence Entropy
GLSZM	Zone Entropy
GLSZM	Large Area High Gray Level Emphasis

NGTDM=Neighborhood Gray Tone Difference Matrix; GLDM=Gray Level Dependence Matrix; GLDM=Gray Level Dependence Matrix; GLSZM=Gray Level Size Zone Matrix.
